# Accessing and Administering Anticipatory Medications for Community End‐of‐Life Symptom Control: A Qualitative Focus Group Study

**DOI:** 10.1111/jocn.70363

**Published:** 2026-05-19

**Authors:** Matthew Bernstein, Louisa Polak, Stephen Barclay, Simon Etkind, Kristian Pollock, Anna Spathis, Markus Schichtel, Sarah Hopkins, Susannah Browne, Ben Bowers

**Affiliations:** ^1^ Palliative and End of Life Care Group in Cambridge (PELiCam), Primary Care Unit, Department of Public Health and Primary Care University of Cambridge Cambridge UK; ^2^ Cambridgeshire and Peterborough NHS Foundation Trust Cambridge UK; ^3^ Cambridge University Hospital's NHS Foundation Trust Cambridge UK; ^4^ School of Health Sciences University of Nottingham Nottingham UK; ^5^ Queen's Institute of Community Nursing London UK

**Keywords:** community health services, general practice, injection, palliative care, pharmacies, subcutaneous

## Abstract

**Aim:**

To understand healthcare professionals' perspectives of what works well and what can be improved in the supply and administration of anticipatory medications at the end of life in the community.

**Design:**

Qualitative interpretive study using focus groups.

**Methods:**

Semi‐structured focus groups included healthcare professionals with experience of using anticipatory medications, and public contributors with lived experiences of relatives' end‐of‐life care. Participants' demographic information was elicited in a brief questionnaire. Transcripts were analysed inductively using thematic analysis. Data were collected in September 2022.

**Setting and Participants:**

Eight focus groups involved 58 UK‐based participants. Each group included people with a variety of professional roles from diverse geographical areas, and public contributors with relevant lived experiences.

**Results:**

The administration of anticipatory prescriptions was widely perceived to be a valuable intervention, but extensive operational challenges were identified, with three interconnected themes arising from the data: (a) Communication between healthcare teams; (b) Intuitive documentation; (c) Accessibility of medications. Addressing these challenges was perceived to be onerous, particularly for nurses and families.

**Conclusions:**

Operational barriers to the timely and appropriate administration of anticipatory medications risk were perceived as adversely affecting patient care and patients' and families' experiences.

**Implications for the Profession and/or Patient Care:**

System‐level improvements are needed to streamline care processes and ensure equitable, appropriate, and timely access to end‐of‐life symptom control medications in the community.

**Reporting Method:**

This study adheres to relevant EQUATOR guidelines and follows the appropriate Standards for Reporting Qualitative Research (SRQR).

**Patient or Public Contribution:**

Our Public and Clinician Advisory Group helped shape questions and commented on findings. Focus groups included public participants with lived experience of end‐of‐life care in the community.

## Impact

- Current methods for formal communication between healthcare providers are slow and vary by locality; as a result, informal communication (e.g., WhatsApp messaging) is used.
- Unintuitive medication documentation systems are leading to delayed administration of medication, a point of frustration for healthcare providers.
- Nurses and caregivers face considerable burdens and operational obstacles to ensure adequate patient care.

## 1 Introduction

Globally, it is estimated between 51% and 56% of deaths occur at home (Adair 2021). In the UK, the proportion of people dying in their own home has increased from 22.4% to 28.4% between 2013 and 2023 (Department of Health and Social Care 2026). The anticipatory prescribing of injectable symptom control medications is a widely used and internationally recommended practice, intended to minimise delays in the management of end‐of‐life symptoms (Wilson et al. 2016; National Institute for Health and Care Excellence 2015; Safer Care Victoria 2023; Royal College of General Practice and Marie Curie 2026). These medications are typically prescribed for pain, nausea and vomiting, agitation and respiratory tract secretions in the last days of life (Department of Health and Social Care 2026; Royal College of General Practice and Marie Curie 2026).

Anticipatory prescribing and administering of end‐of‐life medications involves several steps. A healthcare professional must first identify a need for a patient to be prescribed anticipatory medications. End‐of‐life care planning tools, such as the Gold Standards Framework (GSF), have become instrumental for identifying patients nearing end‐of‐life, triggering putting in place anticipatory medications in a timely fashion, and structuring ongoing community support (Thomas 2003). A prescription and authorisation chart (detailing the indication and allowable dosages of the prescribed medication) must then be completed. The medications prescribed must then be dispensed and brought to the home to be stored for ready access should symptoms arise.

Given the challenges involved in accurately predicting when a patient is entering their last weeks of life, there is high variability regarding the range of time between when a patient is prescribed anticipatory medication and their death (Bowers et al. 2022a). Late identification of imminent dying and paperwork delays may cause caregivers (family and friends) and healthcare providers to leave a dying person's bedside to obtain medications for symptoms that have arisen.

In the UK, Norway, Canada and several other countries, it is usual for nurses to visit the home to administer injectable medications when clinically appropriate (Bowers et al. 2022a; Wilson et al. 2015; Staats et al. 2018; Wowchuk et al. 2009). Nurses can only administer medications if a valid written prescription and authorisation chart is present in the home and can then only use the dose ranges prescribed (Khalil et al. 2019). In the absence of anticipatory medications being already in place, the nurse, patient, or caregiver must: (1) call the general practitioner (GP) practice, out of hours doctor, or emergency services to obtain a clinical assessment; (2) source a prescription and authorisation chart; (3) ensure that the prescription reaches a pharmacist who can dispense it quickly; (4) collect the medication; (5) take it to the patient; and finally arrange for a qualified clinician to administer the medication (Bowers and Redsell 2017). This sequence of tasks is labour‐intensive and frequently delays symptom relief (Bowers and Redsell 2017; Bowers et al. 2026). By ensuring that authorisation charts and medication are already present in the home, anticipatory prescribing can minimise delays and facilitate timely access to symptom relief, especially during out‐of‐hours periods (weekends, bank holidays and at night) when there are more limited services available in the community (Wilson et al. 2016, 2015; National Institute for Health and Care Excellence 2015; Safer Care Victoria 2023; Royal College of General Practice and Marie Curie 2026; Thomas 2003; Bowers et al. 2022a, 2026; Staats et al. 2018; Wowchuk et al. 2009; Khalil et al. 2019; Bowers and Redsell 2017; Hardy et al. 2023).

Healthcare professionals currently prescribe and administer anticipatory medications based on policies supported by limited evidence, particularly regarding the lived experience of patients, caregivers, and healthcare providers (Bowers et al. 2026; Hardy et al. 2023; Jansen, et al. 2018; Bowers, Antunes, et al. 2023). Two previous studies investigated dying patients' and their family caregivers' views and experiences of this care (Pollock et al. 2021; Bowers et al. 2022b). These highlighted the difficulties patients and caregivers face in obtaining adequate explanations about the purpose of anticipatory medications, sourcing timely help, and arranging for prescribed medications to be administered. This can involve interacting with numerous healthcare professionals, often working for different provider organisations. Much of the research to date on anticipatory prescribing relates to policy effectiveness and understanding healthcare providers' perspectives on the challenges and approaches used in prescribing anticipatory medications (Wilson et al. 2015; Pollock et al. 2021; Lawton et al. 2012; Bowers et al. 2020).

Our study focussed on what happens after a prescription has been written, aiming to understand healthcare professionals' perspectives of what works well and what could be improved in the supply and administration of anticipatory medications at the end of life in the community.

## 2 Methods

### 2.1 Design

This interpretive descriptive qualitative study investigated UK‐based stakeholders' perceptions of anticipatory prescribing (Sandelowski 2000). We used a social constructionist perspective, which understands accounts and shared meanings to be shaped by lived experiences and social engagement (Berger and Luckmann 2011). Focus groups were held in person, and facilitators steered the conversation to generate nuanced insights, as stakeholders with similar and diverse experiences interacted with one another (Kevern and Webb 2001).

## 3 Ethics Statement

The University of Cambridge Psychology Research Ethics Committee approved the study [PRE.2022.063]. The focus group facilitators, recognising the sensitive nature of the topics discussed, encouraged a safe space and emphasised that participants could leave the session at any point (none withdrew from the study). Participants were assured that the information shared during the focus groups would not go beyond the immediate research team unless there were concerns that non‐disclosure would result in harm to patients or others.

### 3.1 Recruitment Procedure

Focus groups were conducted as part of a free, one‐day, in‐person conference titled ‘Anticipatory Prescribing in Community End of Life Care: sharing practice developments and research’, held on 21 September 2022 in England (Data S1). Invitations to the conference were disseminated to healthcare professionals across disciplines and backgrounds via national community nursing and palliative care newsletters, X (Twitter) and Facebook, and emails to the PELiCam research group's national contacts. Recipients were asked to share the information with colleagues, leading to a snowball sample of interested healthcare professionals. Public contributors (caregivers, community representatives, and those with an interest in anticipatory prescribing already known to the research group) were also invited to participate.

**FIGURE 1:**
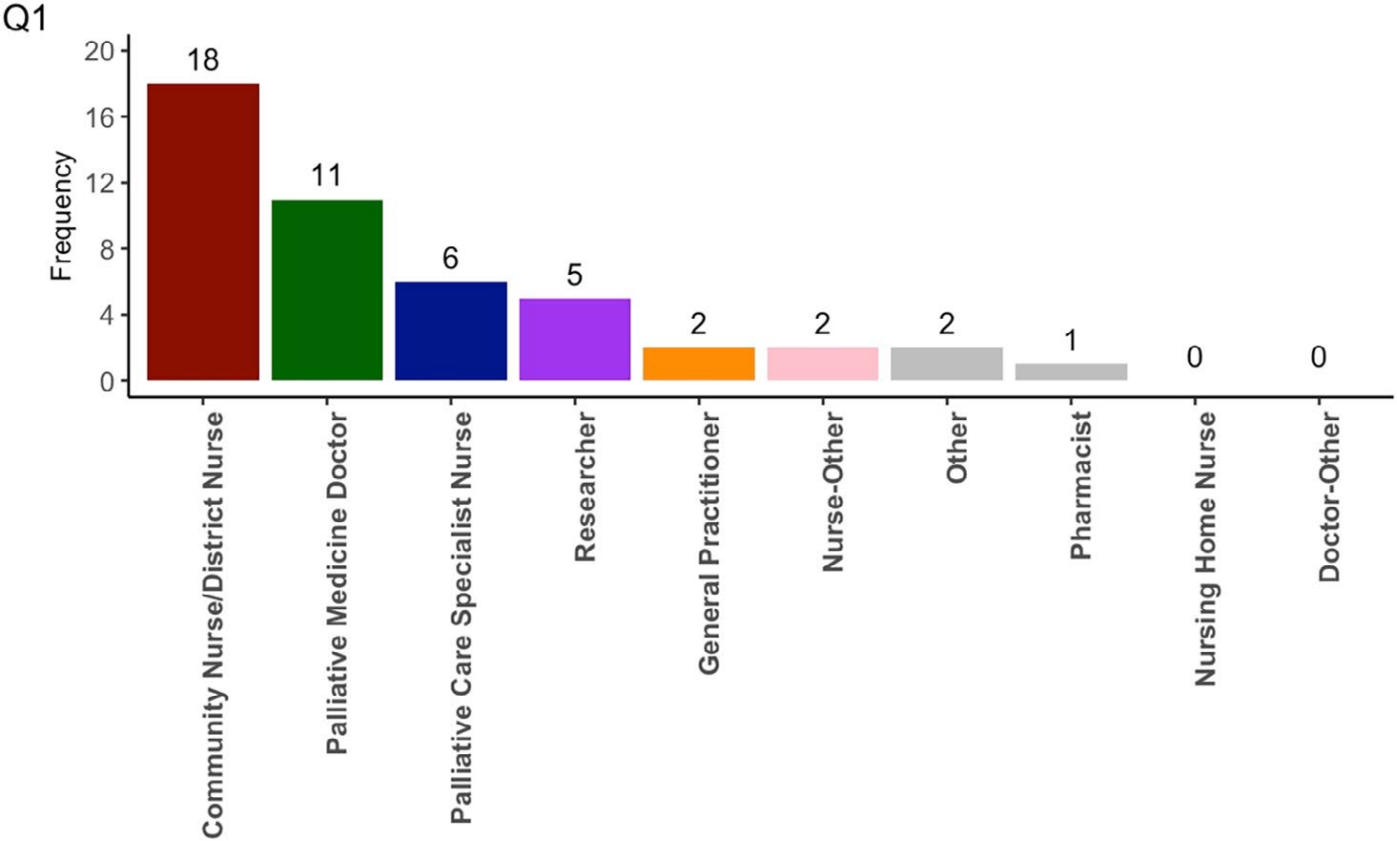
Professional roles of focus group participants. Frequency of responses to anonymous survey question 1 (‘What is your current professional role?’). The most common professional role was identified as ‘Community Nurse/District Nurse’ (*n* = 18) and the second most as ‘Palliative Medicine Doctor’ (*n* = 11). [Colour figure can be viewed at wileyonlinelibrary.com]

**FIGURE 2:**
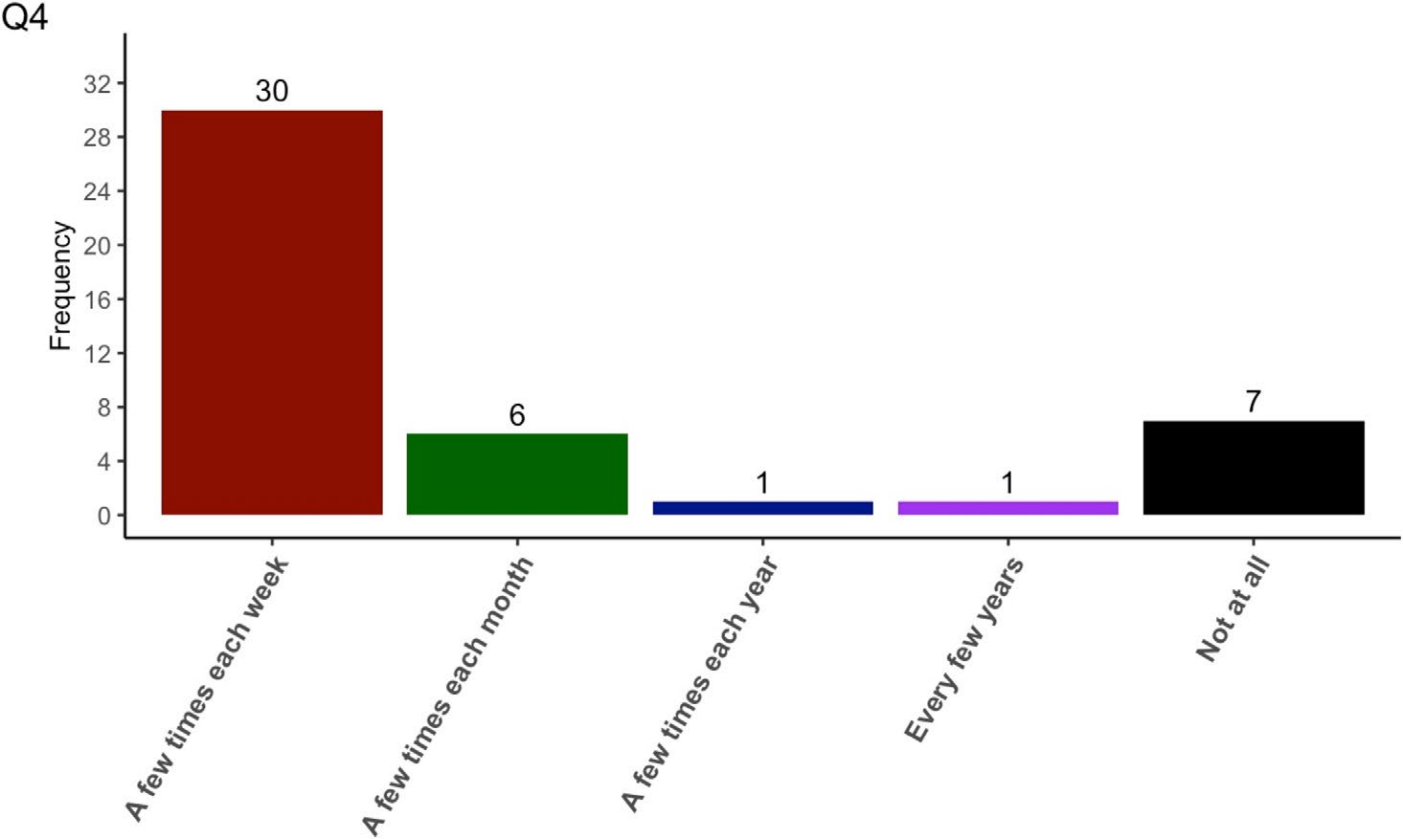
Participants self‐reported involvement in the administration of prescribed anticipatory medications: roles of focus group participants. Frequency of responses to anonymous survey question 2: ‘How often are you involved in processes to use prescribed anticipatory medications?’ [Colour figure can be viewed at wileyonlinelibrary.com]

The focus group was an optional component of the conference, and all attendees were invited to participate in the study ahead of the day. Interested attendees completed a consent form and a short paper questionnaire (Data S2) which covered participants' experiences with anticipatory prescribing, workplace roles and settings and views regarding anticipatory prescribing. Survey responses (summarised in Figures 1 and 2) were anonymised and were not used to identify who joined which focus group. Nobody who offered to participate was excluded, so some participants did not have direct experience of anticipatory prescribing but were involved in wider systems to support the practice of supplying and administering anticipatory medication.

### 3.2 Data Collection

The conference began with a series of short presentations on service evaluations and recent research projects on anticipatory prescribing (Bowers et al. 2022b), followed by focus groups with attendees. Focus groups were 60‐min in length, and a semi‐structured topic guide sought to stimulate discussion of participants' experiences of what works well, challenges, and areas requiring improvement (Data S3). Facilitators asked their group to focus primarily on dispensing, collection and administration of medications, rather than on prescribing.

Eight focus groups, each with five to eight participants, were judged as likely to provide rich and detailed discussions and accounts for the subsequent analysis. This a priori decision was guided by the principles of information power (Malterud et al. 2016). Participants were allocated to focus groups with the intention of achieving a mixed composition of professional roles and geographical locations within each group, and some public contributors with relevant lived experience. Public contributors were family carers. Each group was facilitated by one or more experienced researchers and was digitally audio‐recorded with participants' consent.

### 3.3 Analysis

Focus group recordings were professionally transcribed verbatim. These were checked for accuracy and anonymised by MB. Transcripts were analysed inductively in NVivo 12 using Braun and Clarke's six phases of reflexive thematic analysis: data familiarisation; generating initial codes; constructing themes; reviewing potential themes; defining and naming themes; and refining through writing (Braun and Clarke 2006, 2013).

First, MB familiarised himself with the dataset. To aid rigour and reflexivity, MB and LP coded sections of two transcripts independently, using constant comparison techniques, and MB refined the preliminary inductive coding frame through subsequent discussions with LP, SB and BB. These iterative steps enriched and helped develop MB's coding and interpretative analysis. Patterns and differences in participants' shared experiences and accounts were recorded in visual maps for each focus group to aid analysis. Provisional categories and themes were developed by grouping and organising related codes, with a focus on what was most significant and important to participants, and where accounts diverged. Provisional themes and their boundaries were revised through reflexive discussions with LP, SB and BB, with a range of diverse experiences as clinicians and social scientists (Table 1). With each new discussion, MB then visually mapped categories and developing themes across the dataset. Throughout these iterative steps, we went back to the transcripts to ensure interpretations were reflected and grounded in the primary data.

**TABLE 1:** Authorship positionality.

Author	Positionality/Experience
Matthew Bernstein	MB brings a non‐clinical, non‐NHS, academic healthcare perspective. He has a health psychology background and represents a white, middle‐class demographic.
Dr Louisa Polak	LP brings a GP and academic qualitative research perspective. She has health sociology background and represents a white, middle‐class demographic.
Professor Stephen Barclay	SB brings a GP, palliative medicine and applied healthcare researcher background. He represents a white, middle‐class demographic.
Dr Ben Bowers	BB brings a palliative care, community nurse, healthcare systems design and social science research perspective. He represents a white, middle‐class demographic.

The final themes, the cross‐theme impacts, and the clinical relevance of the findings were refined through discussions between MB, LP, SB, BB, SE, KP, AS, MS, SH and SB. These reflexive discussions, revisiting the primary data and the writing process, helped to refine the final themes.

Survey responses were analysed descriptively (Figures 1 and 2) to understand participants' anticipatory prescribing experiences for the purpose of contextualising themes and aiding in the transferability of findings; images were generated using RStudio (RStudio Team 2020).

We could not always identify the profession of participants from the transcripts, so we excluded this information from our analysis except for when the speaker alluded to their role (e.g., ‘with us as district nurses’). We were unable to reliably differentiate between nurses with differing roles and job titles from the transcripts, so all are referred to as ‘nurses’ in the findings.

## 4 Results

### 4.1 Participant Demographics

There were 58 participants, of whom 47 (81%) supplied demographic information (see survey responses in Figures 1 and 2 and Figures S1 and S5). Most identified as working in community palliative care (*n* = 17) and/or primary care settings (*n* = 21) (Figure S1). Five participants did not identify as working clinically or did not choose one of the provided responses. Most identified as either a community nurse/district nurse (*n* = 18) or palliative care doctor (*n* = 11) (Figure 1). Additionally, two GPs, one pharmacist, and three members of the public took part. Participants had a wide range of experiences concerning anticipatory prescribing in the community: 34 (76%) reported having 11 or more years of experience caring for people approaching the end of their lives (Figure S2).

We were able to identify from the transcripts the working location of 40 participants: 19 (48%) were from East of England (Data S5).

Thirty‐eight participants (84%) who completed the survey reported having direct involvement in processes to use prescribed anticipatory medication. Seven participants (16%) did not report having direct involvement but had interest in their appropriate and timely use (Figure 2).

### 4.2 Thematic Findings

Participants were asked open questions about their experiences with using and administering anticipatory medications. The group discussion focused on ways of ensuring that prescriptions and authorisation charts needed to use the medication were appropriately written, and that community pharmacies were able to dispense medication promptly. Participants identified three elements of practice as key to facilitating the timely administration of anticipatory medication: communication between healthcare professionals, intuitive documentation, and accessibility of medications. While comments about problems predominated, participants also mentioned their local experience of solutions to these problems.

#### 4.2.1 Communication Between Healthcare Teams

Nurse and GP participants reported having large caseloads of patients with ever‐changing needs. They identified that good communication within and between healthcare teams was essential for coordination of patient care: it allowed providers to collaborate and to obtain advice from experienced and trusted colleagues, enabled authorisation forms to be completed promptly and correctly, and helped with sharing those forms with appropriate healthcare teams.

It's good communication between all the different services […]. (Focus Group 1)

Several participants spoke of the difficulties experienced in contacting GPs, which commonly involved calling a GP surgery and waiting in the same telephone queue as patients to speak to a GP. Participants spoke favourably about healthcare provider‐specific ‘bypass’ numbers, which expedited communication with GPs, but this was not available in all GP surgeries.

If you physically need to ring a GP to get some support, we're just sitting there, you're fourteenth in the queue […]. (Focus Group 7)

But they're not always keen to give out backdoor [bypass] numbers even to specialist palliative care. I've got one GP surgery that's given me a backdoor number. I've asked others and they're non‐forthcoming. (Focus Group 7)

An alternative method of sourcing medications and medical input was to send an email to the surgery, expecting that the practice administrative staff, who lack formal medical training, would identify the clinical importance of the email, and relay it promptly to a GP. Several participants described this process as potentially unreliable, causing delays in sourcing medication supplies and access to senior clinical advice:

We have to rely on the very, very busy GP admin staff reading her [hundreds of] emails every morning and working out which one from the district nurse is important and they don't have any medical knowledge […]. (Focus Group 2)

Some nurse and GP participants described that they had set up their own communication routes with each other; informal and unofficial methods of communication that allowed for quick, direct interactions. These informal adaptions included sharing personal work mobile numbers to call or message (e.g., via WhatsApp), rather than single point of access phone numbers for teams:

We like to have that communication with the GPs, they've all got my own number, they've all got my WhatsApp, I get GPs WhatsApping me ‘[…] what do you think about this?’ (Focus Group 7)

However, several participants commented that these informal communication methods were not always faster and raised concerns about data security and personal‐professional boundaries. Participants placed importance on establishing systems that allow healthcare professionals to communicate promptly with one another about patient care, while protecting their personal/professional boundaries and maintaining patient confidentiality.

#### 4.2.2 Intuitive Documentation

The administration of anticipatory medication requires clinicians to have up‐to‐date clinical information. Participants identified that the complexity of paper and electronic medication authorisation charts for anticipatory medications, including checking that all signatures were in place, increased the risk of mistakes and slowed down the timely administration of medications.

But when you've got a chart that you've got to date, sign and print in what is it, nine different places and there's not room and you're doing it in a rush, the chances of you missing a signature. (Focus Group 2)

No nurse in their right mind would have designed that [written prescription and authorisation chart]’. People up here [non‐clinical administrators] have designed it. (Focus Group 7)

Participants expressed concern and frustration that documentation mistakes slowed down the process of medication administration and risked clinical harm. They described having to ask GPs to re‐write prescriptions and authorisation charts and reported that documentation systems were unintuitive and complex to complete. At times documentation errors prevented nurses from being able to administer medications when needed, causing avoidable patient suffering. Some teams had nurse prescribers that could address these problems. In an already‐stretched healthcare system, re‐doing a task was frustrating and strained working relationships:

It [the prescription and authorisation chart] might only be a little bit incorrect but incorrect, […] a tremendous amount of our work is rejecting them, and then what happens is we just go out and do it. (Focus Group 2)

Given their familiarity with documentation procedures, some palliative care specialist participants felt that they were able to offer nurses a better and more responsive service than GPs:

We've got a lot of district nurses actually bypassing GPs and coming to specialist palliative care because GPs won't write it correctly or we're more accessible, we're more responsive. (Focus Group 7)

Participants described that recent changes or updates to anticipatory medication documentation systems, written prescriptions, and authorisation charts had caused problems. Changes to these areas had been implemented at pace during the Covid‐19 pandemic: training took time, and the new systems did not always work at first. Once embedded, the systems were reported as having improved their work and continuity of care. Most participants described a medication prescription and authorisation chart system favourably when it saved them time by reducing mistakes and speeding up the process:

I found at first it was a nightmare [when implemented], but after actually we took a lot of training, we did a lot of training with GPs, and actually it's really effective. […] And it's then put straight onto the patients' notes onto SystmOne [electronic health records] […] and then the drugs obviously get sent to the chemist and then, yeah, so that's one good thing is the [county‐wide, cross‐organisation documentation system], I find it really helped. (Focus Group 6)

Several participants discussed the benefits of SystmOne, an electronic health record system with an integrated cross‐organisation prescription and authorisation chart. Such county‐wide systems were appreciated for their simplicity and the ability to allow different healthcare providers to make changes quickly, automatically updating the patient's health record and making it visible to all community healthcare teams involved:

Before we'd have to go into the surgery and get the drug administration chart written up and signed before you could take out to your patient and then give the care, whereas on SystmOne since Covid we've been able to transcribe from SystmOne from what's been prescribed, so we've been able to do our own drug documentation, so that speeded up the whole process. (Focus Group 5)

The use of different documentation systems in neighbouring geographical areas was problematic, and participants described cases where healthcare providers in one area would not recognise a different area's prescription and authorisation chart or documentation system, hindering care provision. Inter‐regional differences could lead to mistakes in administering medications when patients or healthcare professionals moved between community organisations or care settings.

Participants identified the importance of having medication and prescription infrastructures that are intuitive and dynamic, minimising errors and helping them provide timely patient care. Examples of systems that had achieved this were discussed, speaking particularly positively about moves to paper‐lite systems.

#### 4.2.3 Accessibility of Medications

Difficulties sourcing medication was a key barrier to timely administration that was frequently raised. Once prescribed, anticipatory medications needed to be dispensed and then transported (along with the written prescription and administration chart) to the patient's home. These processes were widely identified as causing problems and delays, particularly pharmacies being a long distance away, limited opening hours, and inadequate stock levels:

We often in [name of town] will have one pharmacy over the weekend that people can go and get medications from and that might be an hour, an hour and a half away. (Participant 1, Focus Group 1)

You've got to travel a very long way some, and you have to phone around all the pharmacies that are open to try and find ones but obviously they [don't] deliver. (Participant 2, Focus Group 1)

When patients entered the dying phase before anticipatory medications were in place, caregivers and providers often needed urgently to source medications. This was particularly difficult in evenings, overnight, weekends, and bank holidays, when few community pharmacies were open.

[…] this is going to be a problem because it's Thursday and it's a bank holiday Monday, and [all laugh] pharmacies going to be closed. (Focus Group 3)

But you can't get hold of the drug because none of the chemists locally are either open or stock it. (Focus Group 5)

Participants spoke positively about having key pharmacies that are always stocked with end‐of‐life medications to aid in timely medication sourcing, although these could be some distance away. There were moving accounts of patients dying while their family was out collecting medications. At times, where patients did not have a family or caregiver able to collect medications, nurses reported delivering the prescribed medication to the home themselves:

However, if I go to a 95‐year‐old person that's living on their own and they've got no family, I'm going to go to the pharmacy and get it and bring it back. […] if that means I've got to drive ten miles to a pharmacy, I'm going to go and do it. (Focus Group 3)

Inadequate systems for obtaining anticipatory medications from the pharmacy was reported to be a major challenge to providing timely, effective symptom control at home. An urgent need for improvements to the infrastructure that supported the dispensing and delivery of medication were highlighted.

## 5 Discussion

Caring for people at the end of life requires evidence‐based best practice. While growing evidence supports clinicians' decisions about what to prescribe and when (Royal College of General Practice and Marie Curie 2026; Bowers and Redsell 2017; Bowers et al. 2022b), there is a knowledge gap concerning the complex processes through which the prescribed medications reach the patient's bedside and are administered when needed. Our study contributes to filling this gap; we highlight problems with communication, documentation and dispensing that act as multi‐faceted obstacles to the timely administration of end‐of‐life symptom control medication, and the creative ways in which these obstacles are effectively circumvented in some places. The concerns and successes in community end‐of‐life care identified in this study are transferable to other care settings, nationally and internationally, and could serve as a model to better understanding gaps in care elsewhere.

Our study provides multi‐voice insights into interrelated cross‐organisational obstacles and the ways healthcare professionals adapt care to address these difficulties. Alongside the unique perspective of what different healthcare professionals identified as challenges in successful anticipatory prescribing, we posited factors that might be underlying these challenges and suggested areas for improvement. We propose that our three key themes of challenges with communication between healthcare teams, intuitive documentation and medication accessibility are connected and are symptomatic of underlying challenges with communication and continuity of end‐of‐life care in the community (National Institute for Health and Care Excellence 2015; Bowers et al. 2022b, 2020).

Previous research indicates that ineffective communication between healthcare professionals contributes to poorer quality and less safe patient care (Ratna 2019). Effective communication is key to effective anticipatory medication use (Wilson et al. 2015; Staats et al. 2018). Adequate end‐of‐life symptom control care cannot be provided if professionals cannot quickly and effectively communicate and coordinate care (Thomas 2003; Wilson et al. 2015; Staats et al. 2018; Bowers et al. 2020; Khalil et al. 2022). This coordination involves discussing, communicating and documenting patients' care plans in a timely and effective way using telephone, electronic or paper systems. Our study participants spoke positively about the expertise of palliative care specialists, whose skills are often relied upon informally to address gaps in access to anticipatory medications. This suggests an opportunity to review the formal roles of specialists to better reflect the increased responsibilities they are assuming informally in practice. A professionals‐only GP practice telephone number is a welcome but not universally available aid. Further research should focus on the effectiveness of current communication methods and potential alternatives (Enzinger et al. 2021; Kripalani et al. 2007).

The variability in required documentation methods between areas is a continuing difficulty, especially as multiple healthcare providers are often involved. Consistent documentation of patients' care is internationally agreed to be a way to provide informational continuity of care and reduce errors that can result in patient harm (Azarm‐Daigle et al. 2015; Yang et al. 2019). Other studies support our finding that the lack of uniformity regarding cross‐organisational clinical documentation strains relationships between colleagues and creates uncertainty over what constitutes a valid anticipatory medication prescription and authorisation chart (Wilson et al. 2016; National Institute for Health and Care Excellence 2015). One potential system‐level intervention would be the introduction of an electronic health records sharing platform to facilitate the rapid transfer of up‐to‐date patient prescription and administration charts across organisations and localities. Our study participants' positive perceptions of the electronic patient records of SystmOne, and other county‐wide cross‐organisation documentation systems used in the UK, indicate potential for operational improvements in supporting rapid, accurate prescription and authorisation documentation (Antunes et al. 2024; Campling et al. 2022). The importance of consistent and clear documentation is highlighted in our UK‐based study, providing inspiration for operational cross‐organisational improvements in international localities facing similar challenges and resource constraints. These issues are likely to be more complex and challenging in countries with there is no unified health service (Khalil et al. 2022; NHSBSA 2023).

Community pharmacies are the primary source of medications for community end‐of‐life care (Valliant et al. 2022; Manolakis and Skelton 2010; Latter et al. 2022; Bowers, Howard, et al. 2023). Our study confirms previously identified challenges with accessing these medications from pharmacies (Khalil et al. 2019) even when prescribing anticipatory medication ahead of clinical need. Limited opening hours and inadequate stocks of palliative medications are a continuing problem that requires urgent attention (NHSBSA 2023; Latter et al. 2022; Bowers, Howard, et al. 2023). Potential system‐level interventions could include funding for medication delivery services and/or incentivising community pharmacies to increase their opening hours. Late identification of the clinical need for anticipatory medications or late recognition of the dying phase (Bowers et al. 2022a; Lawton et al. 2012) might lead to end‐of‐life symptoms arising before anticipatory medications are in a patient's home. Participants in our study highlighted the importance of access to pharmacies stocked with end‐of‐life care medications and suggested that expanded access in geographical areas could address medication sourcing challenges. Some nurse participants in our study described taking on sourcing medications from a pharmacy and collecting them. The frequency with which this is occurring, and its impact on wider care, needs further investigation. Community pharmacies are a keystone for facilitating timely end‐of‐life symptom management care in the community within the UK and internationally: improvements to community‐based care need to look to the role of community pharmacies to improve outcomes.

### 5.1 Strengths and Limitations

Our study recruitment approach ensured a diverse range of healthcare professional participants. The transferability of our findings is strengthened by the range of healthcare voices and clinical expertise included, especially community nurses whose voice is often under‐represented in end‐of‐life care research (Bowers and Redsell 2017; Barclay et al. 2019).

All participants had an interest in anticipatory prescribing and end‐of‐life care, and most had experience in providing this care. They worked in a wide range of areas, serving both urban and rural populations and covering areas with marked economic diversity. Participants' expertise and the range of roles and contexts in which they worked were strengths, allowing us to build a detailed picture of what works in this area of practice and what causes difficulties. The credibility of this picture was enhanced by our use of focus groups, which stimulated discussions and debate between participants as well as eliciting individual accounts (Kevern and Webb 2001).

A minority of participants reported being concerned with unsafe practice in anonymous questionnaires; no patient safety issues were raised in focus group discussions beyond reference to unofficial inter‐team communication methods using their work mobiles. Participants were signposted to their own organisations with regard to data sharing through these unofficial routes.

As well as strengths, focus groups have their own limitations: while group facilitators emphasised that there were no ‘right answers’, some participants may have been hesitant to share their views if they disagreed with others' perspectives (Kevern and Webb 2001; Berger and Luckmann 2011). Further, it was not possible to identify each speaker from the recordings, so we could not identify the profession of the people speaking. The limited ethnic diversity of participants is another potential limitation, as is the selection bias inevitable in any such study: individuals with an interest in palliative care were disproportionately likely to choose to participate.

## 6 Conclusion

Multiple interrelated operational barriers impede the timely supply and administration of anticipatory medications. The current methods for communication between healthcare providers are often slow and rely on formal and informal communication methods that vary between localities. Unintuitive and complex medication documentation systems create delays in accessing and administering medications. Timely dispensing of medication from community pharmacies, especially out‐of‐hours, remains an unresolved challenge in many areas.

Future system‐wide improvements should prioritise robust communication methods between healthcare teams, more responsive and intuitive patient care documentation with minimal regional variation, and expanded anticipatory medication pharmacy dispensing services to ensure high‐quality end‐of‐life symptom management.

## Funding

This work was supported by the Wellcome Trust [225577/Z/22/Z] and the RCN Foundation Professional Bursary Scheme. BB and SB were supported by the NIHR Applied Research Collaboration East of England (NIHR ARC EoE) at Cambridgeshire and Peterborough NHS Foundation Trust. The views expressed are those of the authors and not necessarily those of the NIHR or the Department of Health and Social Care.

## Conflicts of Interest

The authors declare no conflicts of interest.

## Supporting information


**Data S1:** Supporting information.

**Data S2:** Supporting information.

**Figure S1:** Clinical working settings of participants.
**Figure S2:** Years of experience caring for people approaching end of life.
**Figure S3:** Participant involvement in community‐based anticipatory medication clinical governance.
**Figure S4:** Confidence that decisions to use prescribed anticipatory medications are done well in the geographical area of participants' work.
**Figure S5:** Concern over unsafe practices regarding administering anticipatory medication in participants' localities.

**Table S1:** Participants' NHS geographic regions.

## Data Availability

The data that support the findings of this study are available on request from the corresponding author. The data are not publicly available due to privacy or ethical restrictions.

## References

[jocn70363-bib-0001] Adair, T. 2021. “Who Dies Where? Estimating the Percentage of Deaths That Occur at Home.” BMJ Global Health 6, no. 9: e006766. 10.1136/bmjgh-2021-006766/. PMC8420738 34479953

[jocn70363-bib-0002] Antunes, B. C. P. , B. Bowers , S. Barclay , J. Gallagher , R. Conci , and L. Polak . 2024. “Community‐Based Anticipatory Prescribing During COVID‐19: A Qualitative Study.” BMJ Supportive & Palliative Care 14: e2977–e2985. 10.1136/bmjspcare-2022-003597. PMC11672004 35649716

[jocn70363-bib-0003] Azarm‐Daigle, M. , C. Kuziemsky , and L. Peyton . 2015. “A Review of Cross Organizational Healthcare Data Sharing.” Procedia Computer Science 63: 425–432. 10.1016/j.procs.2015.08.363.

[jocn70363-bib-0004] Barclay, S. , E. Moran , S. Boase , et al. 2019. “Primary Palliative Care Research: Opportunities and Challenges.” BMJ Supportive & Palliative Care 9, no. 4: 468–472. 10.1136/bmjspcare-2018-001653. PMC6923936 30755396

[jocn70363-bib-0005] Berger, P. L. , and T. Luckmann . 2011. The Social Construction of Reality: A Treatise in the Sociology of Knowledge. Open Road Media Integrated Media.

[jocn70363-bib-0006] Bowers, B. , B. C. P. Antunes , S. Etkind , et al. 2023. “Anticipatory Prescribing in Community End‐of‐Life Care: Systematic Review and Narrative Synthesis of the Evidence Since 2017.” BMJ Supportive & Palliative Care 13: e612–e623. 10.1136/spcare-2022-004080. PMC10850730 37236648

[jocn70363-bib-0007] Bowers, B. , S. S. Barclay , K. Pollock , and S. Barclay . 2020. “GPs' Decisions About Prescribing End‐of‐Life Anticipatory Medications: A Qualitative Study.” British Journal of General Practice 70, no. 699: e731–e739. 10.3399/bjgp20X712625. PMC7480177 32895243

[jocn70363-bib-0008] Bowers, B. , S. Gwyn , S. Yardley , et al. 2026. “Learning From End‐of‐Life Injectable Medication Patient Safety Incidents in the Community: A Mixed‐Methods Analysis.” British Journal of General Practice 76, no. 763: e151–e162. 10.3399/BJGP.2025.0106. PMC13044229 41022519

[jocn70363-bib-0009] Bowers, B. , P. Howard , B. Madden , K. Pollock , and S. Barclay . 2023. “Is End‐of‐Life Anticipatory Prescribing Always Enough?” BMJ 381: p1106. 10.1136/bmj.p1106. 37192773

[jocn70363-bib-0010] Bowers, B. , K. Pollock , and S. Barclay . 2022a. “Unwelcome Memento Mori or Best Clinical Practice? Community End of Life Anticipatory Medication Prescribing Practice: A Mixed Methods Observational Study.” Palliative Medicine 36: 95–104. 10.1177/02692163211043382. 34493122 PMC8796157

[jocn70363-bib-0011] Bowers, B. , K. Pollock , and S. Barclay . 2022b. “Simultaneously Reassuring and Unsettling: A Longitudinal Qualitative Study of Community Anticipatory Medication Prescribing for Older Patients.” Age and Ageing 51, no. 12: afac293. 10.1093/ageing/afac293. 36477784 PMC9729004

[jocn70363-bib-0012] Bowers, B. , and S. A. Redsell . 2017. “A Qualitative Study of Community Nurses' Decision‐Making Around the Anticipatory Prescribing of End‐of‐Life Medications.” Journal of Advanced Nursing 73, no. 10: 2385–2394. 10.1111/jan.13319. 28423478

[jocn70363-bib-0013] Braun, V. , and V. Clarke . 2006. “Using Thematic Analysis in Psychology.” Qualitative Research in Psychology 3, no. 2: 77–101. 10.1191/1478088706qp063oa.

[jocn70363-bib-0014] Braun, V. , and V. Clarke . 2013. Successful Qualitative Research: A Practical Guide for Beginners. First published ed. Sage.

[jocn70363-bib-0015] Campling, N. , J. Birtwistle , A. Richardson , et al. 2022. “Access to Palliative Care Medicines in the Community: An Evaluation of Practice and Costs Using Case Studies of Service Models in England.” International Journal of Nursing Studies 132: 104275. 10.1016/j.ijnurstu.2022.104275. 35667146

[jocn70363-bib-0040] Department of Health and Social Care . 2026. “Palliative and End of Life Care Profiles—Data | Fingertips [Internet].” https://fingertips.phe.org.uk/profile/end‐of‐life/data#page/4/gid/1938132883/pat/159/par/K02000001/ati/15/are/E92000001/iid/93476/age/1/sex/4/cat/‐1/ctp/‐1/yrr/1/cid/4/tbm/1.

[jocn70363-bib-0016] Enzinger, L. , P. Dumanoir , B. Boussat , P. Couturier , and P. Francois . 2021. “Direct Phone Communication to Primary Care Physician to Plan Discharge From Hospital: Feasibility and Benefits.” BMC Health Services Research 21: 1352. 10.1186/s12913-021-07398-w. 34922549 PMC8684651

[jocn70363-bib-0017] Hardy, B. , N. King , and A. Rodriguez . 2023. “‘Self’ and ‘Dyadic’ Managing in the Last Year of Life.” Abstracts of the 8th World Research Congress of the European Association for Palliative Care (EAPC) [Internet]. Abstract Number P312. 10.1177/0269216314532748.

[jocn70363-bib-0018] Jansen, K. , D. F. Haugen , L. Pont , and S. Ruths . 2018. “Safety and Effectiveness of Palliative Drug Treatment in the Last Days of Life—A Systematic Literature Review.” Journal of Pain and Symptom Management 55, no. 2: 508–521.e3. 10.1016/j.jpainsymman.2017.06.010. 28803078

[jocn70363-bib-0019] Kevern, J. , and C. Webb . 2001. “Focus Groups as a Tool for Critical Social Research in Nurse Education.” Nurse Education Today 21, no. 4: 323–333. 10.1054/nedt.2001.0563. 11339876

[jocn70363-bib-0020] Khalil, H. , M. Garett , A. Byrne , et al. 2022. “Mapping End‐of‐Life and Anticipatory Medications in Palliative Care Patients Using a Longitudinal General Practice Database.” Palliative & Supportive Care 20, no. 1: 94–100. 10.1017/S1478951521000092. 33750494

[jocn70363-bib-0021] Khalil, H. , P. Poon , A. Byrne , and E. Ristevski . 2019. “Challenges Associated With Anticipatory Medications in Rural and Remote Settings.” Journal of Palliative Medicine 22, no. 3: 297–301. 10.1089/jpm.2018.0354. 30427742

[jocn70363-bib-0022] Kripalani, S. , F. LeFevre , C. O. Phillips , M. V. Williams , P. Basaviah , and D. W. Baker . 2007. “Deficits in Communication and Information Transfer Between Hospital‐Based and Primary Care Physicians: Implications for Patient Safety and Continuity of Care.” Journal of the American Medical Association 297, no. 8: 831. 10.1001/jama.297.8.831. 17327525

[jocn70363-bib-0023] Latter, S. , N. Campling , J. Birtwistle , et al. 2022. “Patient and Carer Access to Medicines at End of Life: The ActMed Mixed‐Methods Study.” Health and Social Care Delivery Research 10, no. 20: 1–208. 10.3310/FIQE5189. 35901229

[jocn70363-bib-0024] Lawton, S. , M. Denholm , L. Macaulay , E. Grant , and A. Davie . 2012. “Timely Symptom Management at End of Life Using ‘Just in Case’ Boxes.” British Journal of Community Nursing 17, no. 4: 182–190. 10.12968/bjcn.2012.17.4.182. 22848942

[jocn70363-bib-0025] Malterud, K. , V. D. Siersma , and A. D. Guassora . 2016. “Sample Size in Qualitative Interview Studies: Guided by Information Power.” Qualitative Health Research 26, no. 13: 1753–1760. 10.1177/1049732315617444. 26613970

[jocn70363-bib-0026] Manolakis, P. G. , and J. B. Skelton . 2010. “Pharmacists' Contributions to Primary Care in the United States Collaborating to Address Unmet Patient Care Needs: The Emerging Role for Pharmacists to Address the Shortage of Primary Care Providers.” American Journal of Pharmaceutical Education 74, no. 10: S7. 10.5688/aj7410s7. 21436916 PMC3058447

[jocn70363-bib-0027] National Institute for Health and Care Excellence . 2015. “Care of Dying Adults in the Last Days of Life [Internet].” NICE Guidance NG31. https://www.nice.org.uk/guidance/ng31. 26741019

[jocn70363-bib-0041] NHSBSA . 2023. “General Pharmaceutical Services in England 2015/16–2022/23 [Internet].” https://www.nhsbsa.nhs.uk/statistical‐collections/general‐pharmaceutical‐services‐england/general‐pharmaceutical‐services‐england‐201516‐202223.

[jocn70363-bib-0028] Pollock, K. , E. Wilson , G. Caswell , et al. 2021. “Family and Health‐Care Professionals Managing Medicines for Patients With Serious and Terminal Illness at Home: A Qualitative Study.” Health Services and Delivery Research 9, no. 14: 1–162. 10.3310/hsdr09140. 34410684

[jocn70363-bib-0029] Ratna, H. 2019. “The Importance of Effective Communication in Healthcare Practice.” Harvard Public Health Review 23: 1–6. 10.54111/0001/W4.

[jocn70363-bib-0030] Royal College of General Practice & Marie Curie . 2026. “The Daffodil Standards: Self‐Assessment Evidence and Guidance [Internet].” https://www.rcgp.org.uk/learning‐resources/daffodil‐standards.

[jocn70363-bib-0031] RStudio Team . 2020. RStudio: Integrated Development for R. RStudio, PBC.

[jocn70363-bib-0032] Safer Care Victoria . 2023. “Strategic Plan 2023–2026 [Internet].” Melbourne: State of Victoria. https://www.safercare.vic.gov.au.

[jocn70363-bib-0033] Sandelowski, M. 2000. “Whatever Happened to Qualitative Description?” Research in Nursing & Health 23, no. 4: 334–340. 10.1002/1098-240x(200008)23:4<334::aid-nur9>3.0.co;2-g. 10940958

[jocn70363-bib-0034] Staats, K. , O. Tranvåg , and E. K. Grov . 2018. “Home‐Care Nurses' Experience With Medication Kit in Palliative Care.” Journal of Hospice and Palliative Nursing 20, no. 6: E1–E9. 10.1097/NJH.0000000000000518. 30379802

[jocn70363-bib-0035] Thomas, K. 2003. Caring for the Dying at Home: Companions on the Journey. Radcliffe Medical.

[jocn70363-bib-0036] Valliant, S. N. , S. C. Burbage , S. Pathak , and B. Y. Urick . 2022. “Pharmacists as Accessible Health Care Providers: Quantifying the Opportunity.” Journal of Managed Care & Specialty Pharmacy 28, no. 1: 85–90. 10.18553/jmcp.2022.28.1.85. 34949110 PMC8890748

[jocn70363-bib-0037] Wilson, E. , H. Morbey , J. Brown , S. Payne , C. Seale , and J. Seymour . 2015. “Administering Anticipatory Medications in End‐of‐Life Care: A Qualitative Study of Nursing Practice in the Community and in Nursing Homes.” Palliative Medicine 29, no. 1: 60–70. 10.1177/0269216314543042. 25070861 PMC4266693

[jocn70363-bib-0038] Wilson, E. , J. Seymour , and C. Seale . 2016. “Anticipatory Prescribing for End of Life Care: A Survey of Community Nurses in England.” Primary Health Care 26, no. 9: e1151. 10.7748/phc.2016.e1151.

[jocn70363-bib-0039] Wowchuk, S. M. , E. A. Wilson , L. Embleton , M. Garcia , M. Harlos , and H. M. Chochinov . 2009. “The Palliative Medication Kit: An Effective Way of Extending Care in the Home for Patients Nearing Death.” Journal of Palliative Medicine 12, no. 9: 797–803. 10.1089/jpm.2009.0048. 19624267

[jocn70363-bib-0042] Yang, C. , T. C. Chou , and Y. H. Chen . 2019. “Bridging Digital Boundary in Healthcare Systems—An Interoperability Enactment Perspective.” Computer Standards & Interfaces 62: 43–52. 10.1016/j.csi.2018.08.001.

