# Supplementary material for: Accessing and Administering Anticipatory Medications for Community End‐of‐Life Symptom Control: A Qualitative Focus Group Study
Source: J Clin Nurs. 2026 May 19;35(9):3921–30. doi: 10.1111/jocn.70363 (PMC13431712; doi:10.1111/jocn.70363)
Supplement: Supplementary file 2 — Data S2: Supporting information. [file JOCN-35-3921-s003.docx]

**Topic guide**

Name of study: Administering anticipatory medications at the end-of-life: what works well and areas in need of improvement

**Instructions for facilitator**

- The workshop round-table discussion (focus group) is 60-minutes in length.
- **Please go through the ‘introduction’** ***at the start of the session***, which will take approximately 5 minutes. ***All of the ground-rules must be highlighted, especially confidentiality and the need to maintain patient/colleague anonymity.***
- 3 topics will be covered within the session, and **it is essential to cover all 3 topics.** We have suggested timings for each topic but please feel free to use your own discretion.
- *It is not essential to cover every prompt under each topic - these are there to help direct and focus the discussion, but you are free to use your own discretion.*
- **Try to let the conversation flow between group members**, rather than interrupting with prompts and probes. Only use prompts if the group is quiet or it is necessary to refocus the discussion.
- **After the focus group, please write a short (1-2 paragraph) reflection about how you felt it went as soon as possible afterwards.** It would be helpful to have your comments on group dynamics or the tone/mood of the session, as well as how you felt as the facilitator. There is a blank page at the end of the topic guide where you can record your reflections. Alternatively, you can just send me an email with your thoughts.
- **Voice recorders: each facilitator will have 2 voice recorders- please use both.** **We’d advise that you press ‘record’ just before the ‘introduction’, so you don’t forget to do so.** Please hand over the recorders after the session: all recordings will be securely downloaded to a laptop at the end of the day.

**Facilitator’s introduction (5 minutes)**

1. **The facilitator introduces self**
2. **The facilitator briefly explains purpose of the discussion:** to explore participants views and experiences of what works well and can be improved in decisions to use (administer) anticipatory medications at the end of life in the community. This is once medications have been prescribed.
3. **The facilitator explains the ground rules for the focus group discussion:**

- We want you to do the talking, one person at a time
- We would ideally like to hear from everyone, but you are free to participate as much or as little as you like
- There are no right or wrong answers: please be sensitive to everyone’s views and experiences.
- What is shared in this room stays in this room: please keep everything you hear confidential
- When discussing examples from your own practice, please be mindful of the anonymity of your patients and colleagues

**4) The facilitator reminds the participants about the audio-recording of the discussions for**

**research purposes and reassures them about confidentiality and anonymity.** If there is anyone

who hasn’t yet signed a consent form and you want to participate, please can you do so now and

place it in this box.

**Round-table discussion (55 minutes):**

**Introduction (5 minutes):**

Ask participants to give introduce themselves by giving their name, role, workplace and their typical involvement in decisions to administer / use anticipatory medications

**Topic 1: What works well (20 minutes):**

**1) Where you practice, what factors seem to promote the appropriate and timely use (administration) of prescribed anticipatory medications?**

- *Ask for examples to illustrate points made*

**2) How much should clinicians involve families in decisions to use (administer) prescribed anticipatory medications?**

**Topic 2: What can go wrong (10 minutes):**

**3) Could you share an example from your practice of when things went wrong or almost went wrong regarding using (administering) prescribed anticipatory medication?**

- *Remind to be mindful of preserving the anonymity of those involved*
- *Move the conversation on to topic 3 after 10-minutes if there are multiple examples given*

**Topic 3: What could be improved (15 minutes):**

**4) Are there elements of systems to use (administer) prescribed medications in your area that could be improved?**

- *Ask for examples to illustrate points made*
- *If limited response, prompt participants to consider:*
  - *Patient and family factors (e.g. preferences, mental capacity, support structures)*
  - *systems factors (e.g. policies/procedures, speed of access to services)*
  - *healthcare professional factors (e.g. experience, training, skills in recognising dying)*
  - *resource/economic factors*

**Close (5 minutes):**

- **Go around the group asking if they have other comments they wish to make**
- **Thank group for participating. Re-iterate confidentiality.**

**Facilitator reflections**

Please write a short (1-2 paragraph) reflection on the next page about how you felt the session went as soon as possible afterwards and return it to Ben. Alternatively, you can just send an email with your thoughts if you’d prefer. You may wish to comment on group dynamics or the tone/mood of the session, as well as how you felt as the facilitator.

**Facilitator reflection:**

**Facilitator name:**

**Group name:**
