# Supplementary material for: Accessing and Administering Anticipatory Medications for Community End‐of‐Life Symptom Control: A Qualitative Focus Group Study
Source: J Clin Nurs. 2026 May 19;35(9):3921–30. doi: 10.1111/jocn.70363 (PMC13431712; doi:10.1111/jocn.70363)
Supplement: Supplementary file 3 — Figure S1: Clinical working settings of participants. Figure S2: Years of experience caring for people approaching end of life. Figure S3: Participant involvement in community‐based anticipatory medication clinical governance. Figure S4: Confidence that decisions to use prescribed anticipatory medications are done well in the geographical area of participants' work. Figure S5: Concern over unsafe practices regarding administering anticipatory medication in participants' localities. [file JOCN-35-3921-s004.docx]

**Supplemental Figures S1-5**

**
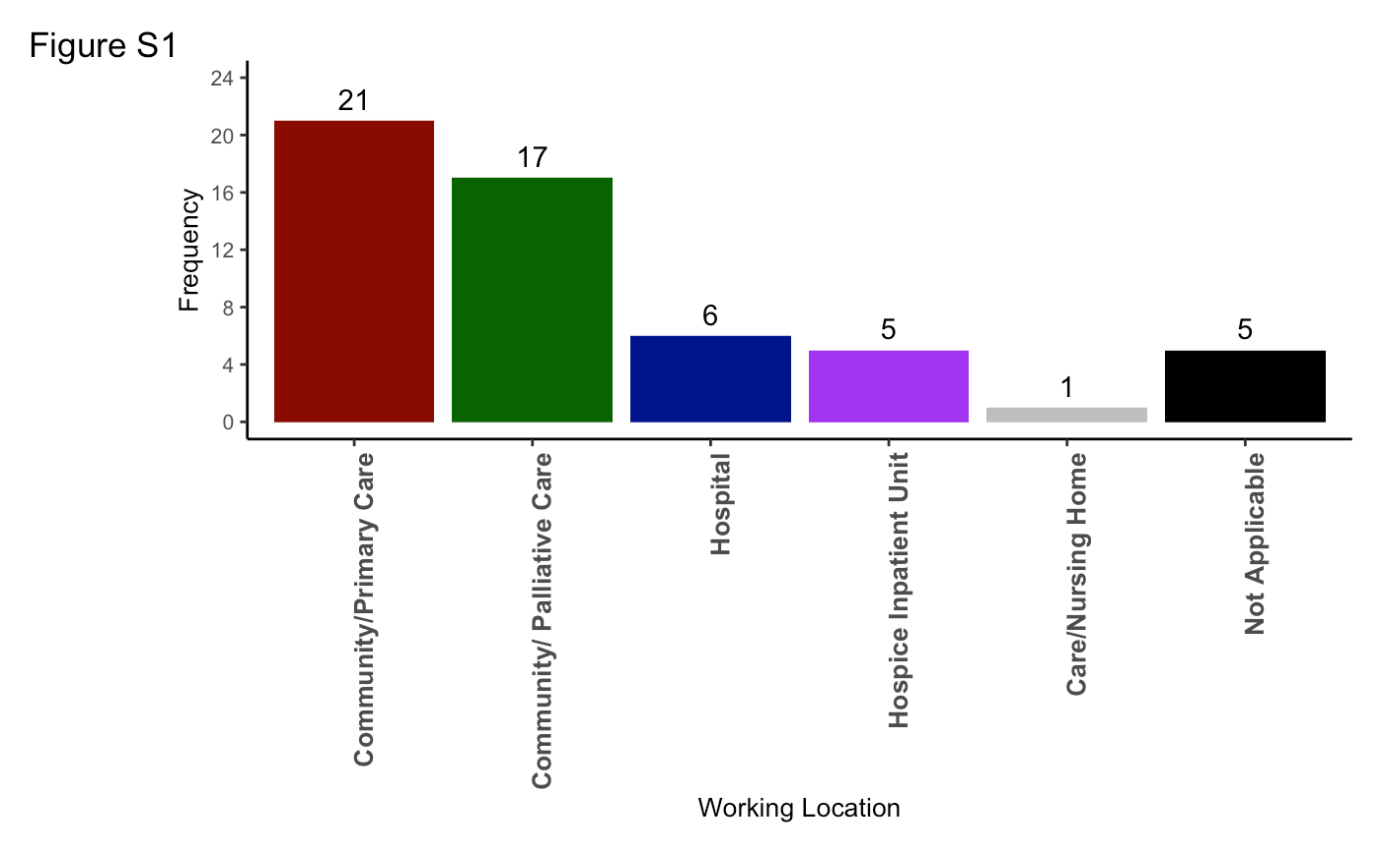
Figure S1.** Clinical working settings of participants

**Figure S1 -** Frequency of responses to anonymous survey question 2 (“If you work clinically, what clinical setting do you work in?”). Most participants identified working in community palliative

(n = 17) and/or primary care settings (n = 21). Some participants (n = 5) did not identify as working clinically or did not choose one of the provided responses.


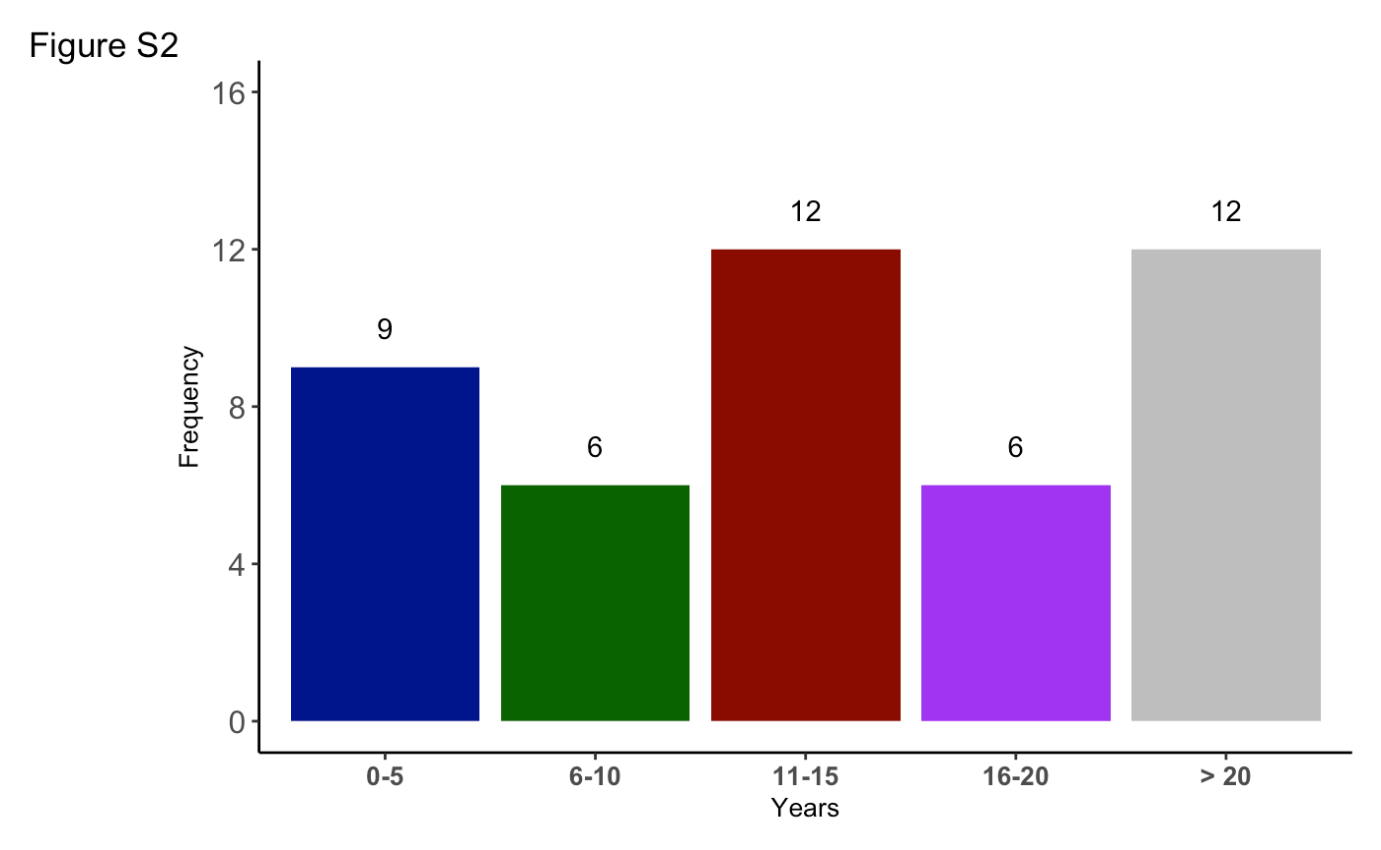
**Figure S2.** – Years of experience caring for people approaching end of life

**Figure S2 -** Frequency of responses to anonymous survey question 3 (“How many years of experience do you have of caring for people approaching the end of their lives?”). Participants spent a wide range of time caring for people at the end of their lives. Thirty-four participants (76%) reported having more than 11 years of experience caring for people approaching the end of their lives.

**
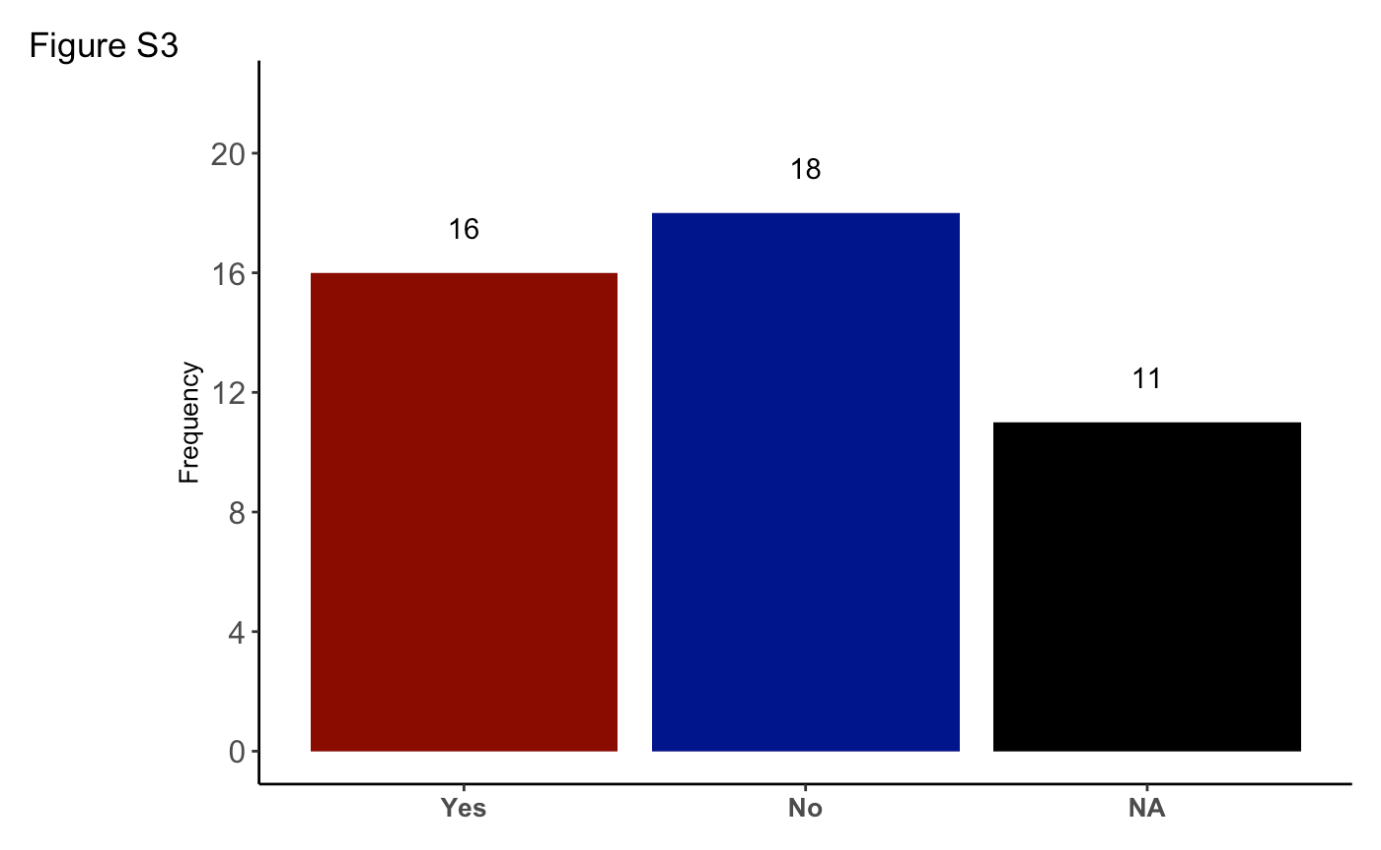
Figure S3.** Participant involvement in community-based anticipatory medication clinical governance

**Figure S3 -** Frequency of responses to anonymous survey question 5 (“Have you been involved in community-based anticipatory medication clinical governance (e.g. writing policies, guidelines, audit etc.)?”). Of the participants who responded, approximately equal numbers of participants identified being involved (n = 16) and not being involved (n = 17) with clinical governance of anticipatory medications. Some participants (n = 11) did not respond to the question.

**
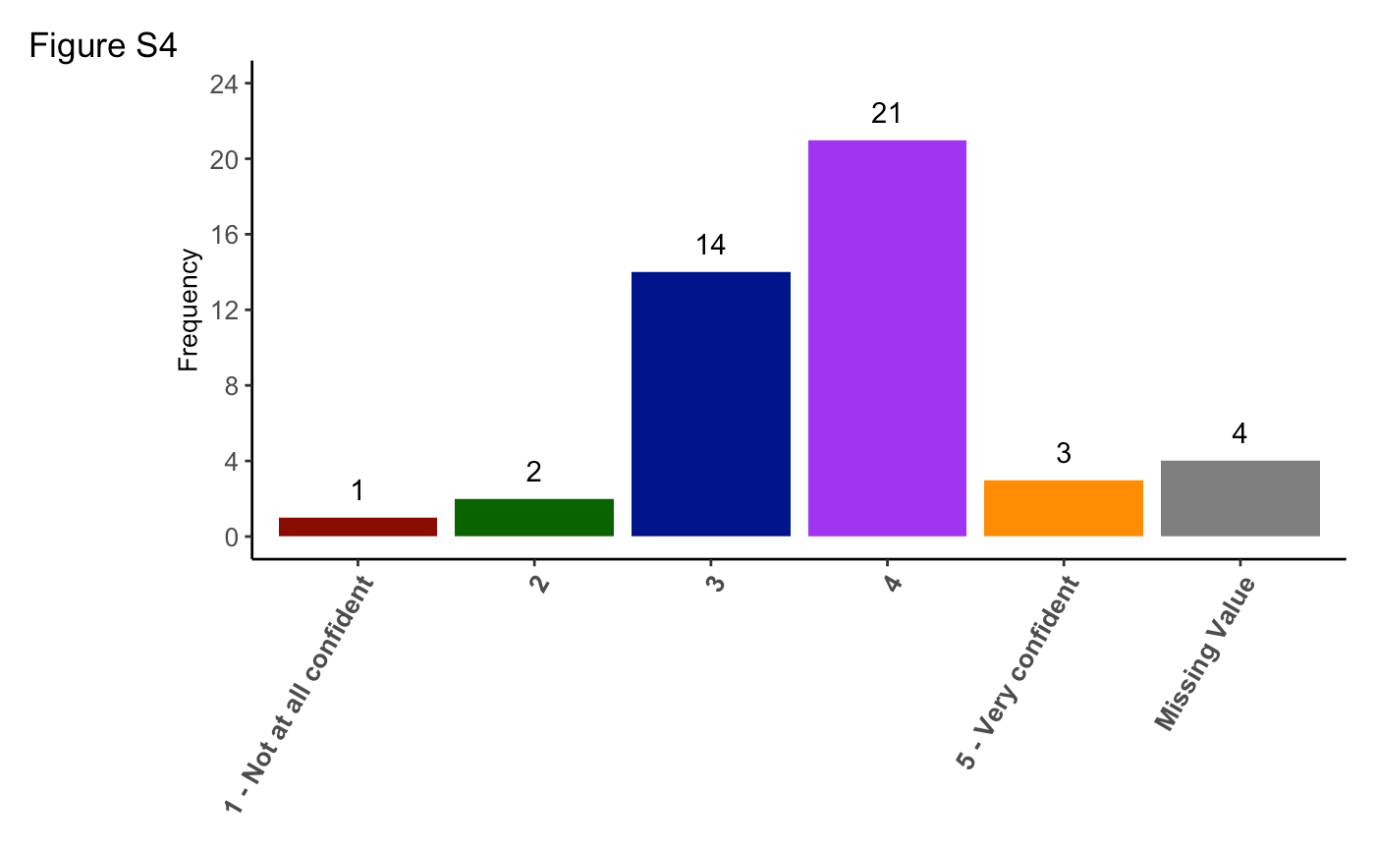
Figure S4.** Confidence that decisions to use prescribed anticipatory medications are done well in the geographical area of participants’ work

**Figure S4 -** Frequency of responses to anonymous survey question 6 (“How confident are you that decisions to use prescribed anticipatory medications are done well in the geographical area you work in?”). Most participants (n = 21) self-identified as slightly confident (‘4’) that decisions to use prescribed AMs are done well in their locality.

**
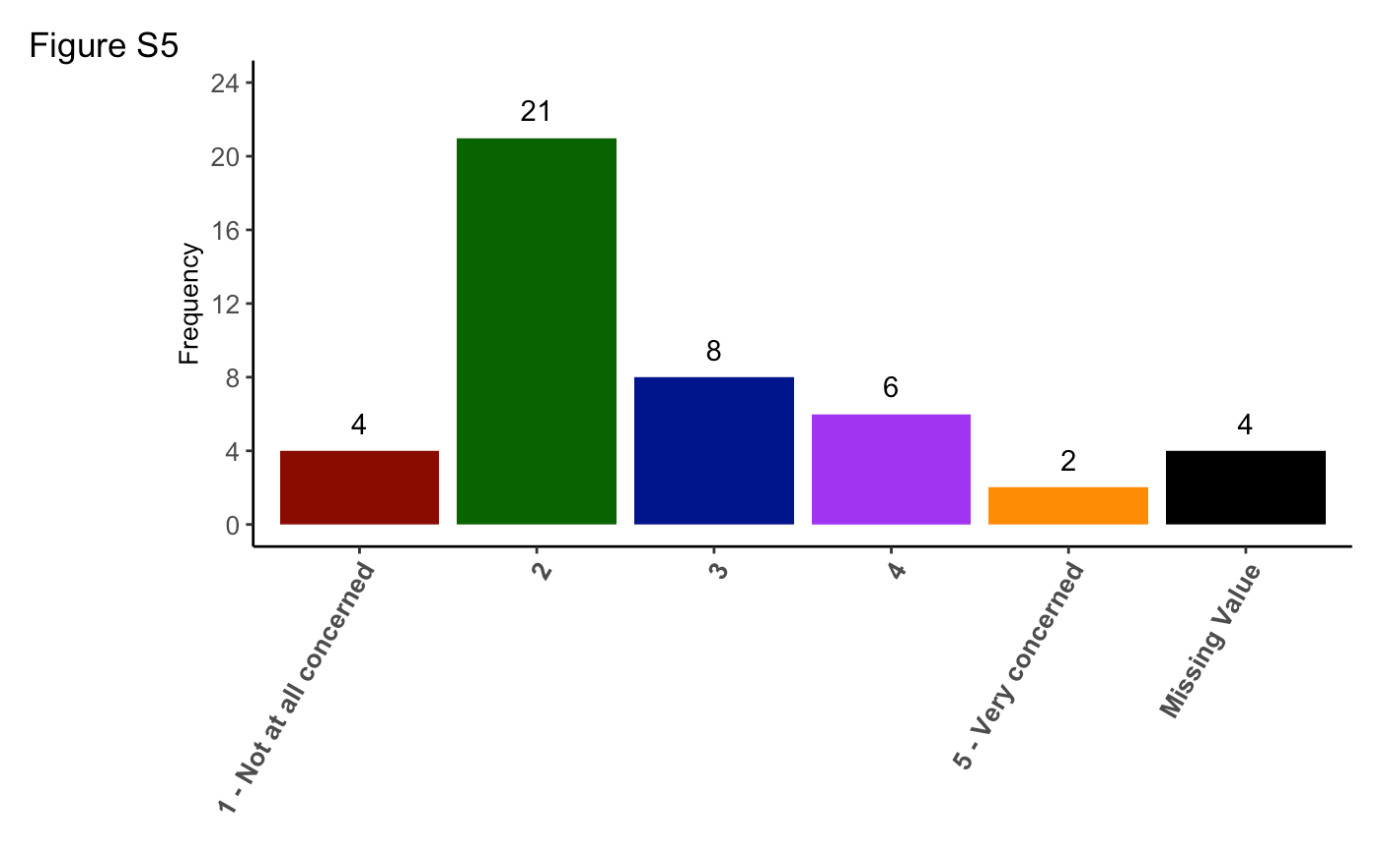
Figure S5.** Concern over unsafe practices regarding administering anticipatory medication in participants’ localities

**Figure S5 -** Frequency of responses to anonymous survey question 7 (“How concerned are you about unsafe practice about administering anticipatory medication in your locality?”). Most participants (n = 21) felt less concerned (‘2’) about unsafe anticipatory prescribing administration practices in their locality.
