# Supplementary material for: Accessing and Administering Anticipatory Medications for Community End‐of‐Life Symptom Control: A Qualitative Focus Group Study
Source: J Clin Nurs. 2026 May 19;35(9):3921–30. doi: 10.1111/jocn.70363 (PMC13431712; doi:10.1111/jocn.70363)
Supplement: Supplementary file 4 — Table S1: Participants' NHS geographic regions. [file JOCN-35-3921-s001.docx]

### **Table 1.** – Participants’ NHS Geographic Regions

| **NHS Geographic Region** | | **Participants** | **%** |
| --- | --- | --- | --- |
| East of England | 19 | | 47.5 |
| London | 8 | | 20 |
| Midlands | 8 | | 20 |
| South West | 2 | | 5.0 |
| South East | 1 | | 2.5 |
| North West | 1 | | 2.5 |
| Wales | 1 | | 2.5 |
| Ireland | 0 | | 0 |
| Scotland | 0 | | 0 |
| North East and Yorkshire | 0 | | 0 |
| **Total** | **40** | | **100** |
| **Not Available** | **18** | |  |

**Table 1**: Geographic region of participants obtained from audio transcripts expressed as number of participants from that region and as percentage of the total. In instances when participants did not mention the region where they worked, they were recorded as “Not available”.
